# Relationship Between Myocardial Strain and Extracellular Volume: Exploratory Study in Patients with Severe Aortic Stenosis Undergoing Photon-Counting Detector CT

**DOI:** 10.3390/diagnostics15020224

**Published:** 2025-01-19

**Authors:** Costanza Lisi, Victor Mergen, Lukas J. Moser, Konstantin Klambauer, Jonathan Michel, Albert M. Kasel, Hatem Alkadhi, Matthias Eberhard

**Affiliations:** 1 Diagnostic and Interventional Radiology, University Hospital Zurich, University of Zurich, CH-8091 Zurich, Switzerland; costanzalisi95@gmail.com (C.L.); victor.mergen@usz.ch (V.M.); lukasjakob.moser@usz.ch (L.J.M.); konstantin.klambauer@usz.ch (K.K.); hatem.alkadhi@usz.ch (H.A.); 2 Department of Biomedical Sciences, Humanitas University, via Rita Levi Montalcini 4, 20090 Pieve Emanuele, Italy; 3 IRCCS Humanitas Research Hospital, Via Manzoni 56, Rozzano, 20089 Milan, Italy; 4 Department of Cardiology, University Heart Center, University Hospital Zurich, University of Zurich, CH-8091 Zurich, Switzerland; jonathan.michel@usz.ch (J.M.); markus.kasel@usz.ch (A.M.K.)

**Keywords:** photon-counting detector CT, myocardial strain, myocardial extracellular volume, aortic stenosis, transcatheter aortic valve replacement

## Abstract

**Background/Objectives**: Diffuse myocardial fibrosis and altered deformation are relevant prognostic factors in aortic stenosis (AS) patients. The aim of this exploratory study was to investigate the relationship between myocardial strain, and myocardial extracellular volume (ECV) in patients with severe AS with a photon-counting detector (PCD)-CT. **Methods**: We retrospectively included 77 patients with severe AS undergoing PCD-CT imaging for transcatheter aortic valve replacement (TAVR) planning between January 2022 and May 2024 with a protocol including a non-contrast cardiac scan, an ECG-gated helical coronary CT angiography (CCTA), and a cardiac late enhancement scan. Myocardial strain was assessed with feature tracking from CCTA and ECV was calculated from spectral cardiac late enhancement scans. **Results**: Patients with cardiac amyloidosis (*n* = 4) exhibited significantly higher median mid-myocardial ECV (48.2% versus 25.5%, *p* = 0.048) but no significant differences in strain values (*p* > 0.05). Patients with prior myocardial infarction (*n* = 6) had reduced median global longitudinal strain values (−9.1% versus −21.7%, *p* < 0.001) but no significant differences in global mid-myocardial ECV (*p* > 0.05). Significant correlations were identified between the global longitudinal, circumferential, and radial strains and the CT-derived left ventricular ejection fraction (EF) (all, *p* < 0.001). Patients with low-flow, low-gradient AS and reduced EF exhibited lower median global longitudinal strain values compared with those with high-gradient AS (−15.2% versus −25.8%, *p* < 0.001). In these patients, the baso-apical mid-myocardial ECV gradient correlated with GLS values (R = 0.28, *p* = 0.02). **Conclusions**: In patients undergoing PCD-CT for TAVR planning, ECV and GLS may enable us to detect patients with cardiac amyloidosis and reduced myocardial contractility

## 1. Introduction

Aortic stenosis (AS) represents one of the most common types of valvular heart disease in Europe and North America [1]. A meta-analysis of studies conducted in Europe, the USA and Taiwan found a prevalence of severe AS of 3.4% in subjects older than 75 years [2].

Diffuse myocardial fibrosis and alterations in myocardial strain have been described in patients with severe AS [3,4,5] and both were identified as independent predictors of morbidity and mortality after transcatheter aortic valve replacement (TAVR) [3,4,6]. The calculation of myocardial extracellular volume (ECV) to quantify myocardial fibrosis is usually performed using cardiac magnetic resonance imaging, while strain analysis is performed by using both magnetic resonance imaging and echocardiography [3,4,7,8,9]. CT imaging has evolved as a standard imaging tool for the pre-interventional assessment of patients with severe AS [10,11], with the latest generation of CT technology enabling advanced myocardial characterization. CT-derived myocardial strain measurements are feasible in patients with advanced cardiac valve disease; they are highly reproducible and correlate with echocardiography strain measurements [12,13,14,15].

Photon-counting detector (PCD) CT facilitates the calculation of myocardial ECV owing to the inherent acquisition of spectral CT data, which renders the separate acquisition and registration of non-contrast CT unnecessary [6,16,17,18]. When using a retrospectively ECG-gated acquisition (i.e., 4D-CT) for coronary CT angiography (CCTA), a CT-based myocardial strain assessment is possible with excellent reproducibility in comparison to echocardiography [5].

The aim of this exploratory study was to investigate the relationship between myocardial strain and myocardial ECV in patients with severe AS with PCD–CT.

## 2. Materials and Methods

### 2.1. Study Population

**Figure 1:**
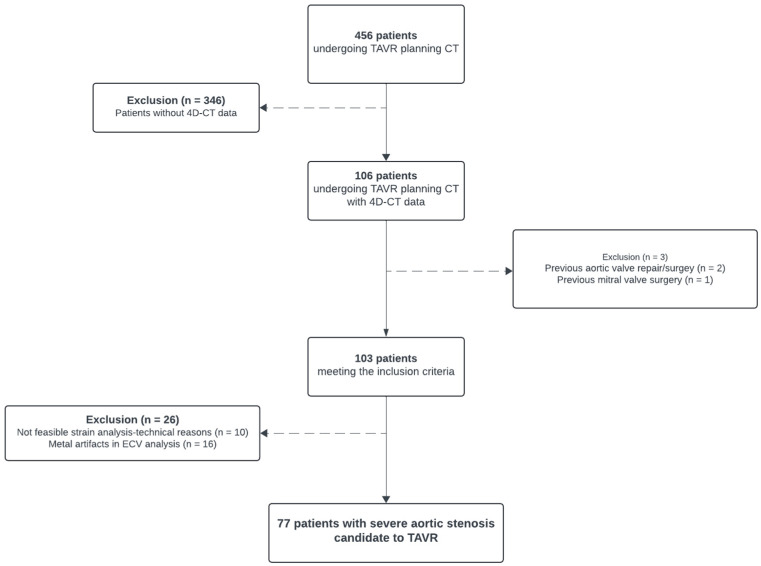
Flow-chart depicting study population inclusion.

This retrospective study was conducted at a tertiary academic hospital and had institutional review board and ethics committee agreement. All subjects provided written general consent for further use of their data for anonymized research. For study inclusion, patients with severe AS undergoing CT for TAVR planning on a dual-source PCD-CT between January 2022 and May 2024 were screened. One hundred and six patients met the inclusion criteria for undergoing a CCTA performed in the ECG-gated retrospective helical mode followed by a cardiac late enhancement (LE) scan. Our exclusion criterion was previous valve surgery (*n* = 3). Moreover, patients with inadequate image quality for ECV or strain calculation were excluded (*n* = 26; 16 patients presenting with severe artifacts in delayed-enhancement scan due to pacemaker leads and in 10 patients the left ventricular [LV] apex was not entirely covered). The study flow-chart is presented in Figure 1.

Patients’ hematocrit (Hct) on the day of CT was collected from the electronic records. Patients with prior myocardial infarction and cardiac amyloidosis were identified based on previous medical records including foregoing MRI, positron emission tomography, and single photon emission computed tomography. Patients were subdivided according to the type of severe AS in line with echocardiographic findings and following international recommendations into patients with high-gradient AS and patients with low-flow, low-gradient AS [19].

### 2.2. CT Data Acquisition and Image Reconstruction

All CT scans were acquired using a first-generation dual-source PCD-CT (NAEOTOM Alpha; Siemens Healthineers AG; Forchheim, Germany). The scan protocol included an ECG-gated non-contrast cardiac scan dedicated to calcium scoring, followed by an ECG-gated CCTA dedicated to assessing the coronary arteries and the left ventricular outflow tract, a high-pitch whole body aortography dedicated to vascular access pathway evaluation, and an ECG-gated cardiac LE scan dedicated to screening for myocardial scars.

CCTA was initiated after the intravenous injection of a weight-based contrast media protocol (60–80 mL iopromidum, Ultravist 370 mg I/mL; Bayer Healthcare, Berlin, Germany) and 20 mL of a saline chaser (NaCl 0.9%) at a flow-rate of 5–6 mL/s. 4D-CCTA was performed in the ECG-gated retrospective helical mode with an ECG-pulsing window fixed from 30% to 80% of the R-R interval.

The cardiac LE scan was acquired 5 min after contrast media administration in the prospective ECG-triggered sequential mode with an ECG window fixed to an absolute RR interval of 280 milliseconds, as previously shown [16]. The gantry rotation time was 0.25 s for all ECG-gated scans.

### 2.3. Strain Analysis

For the strain analysis, retrospectively acquired ECG-gated 4D-CCTA were reconstructed in 10% steps of the cardiac cycle. A feature-tracking based strain analysis was performed with a dedicated software (Medis CT suite, version 1.4.0.158, Medis medical Imaging, Leiden, The Netherlands) by a blinded member of the study team with 4 years of experience in cardiac imaging (C.L.). The 3D viewer Medis tool was used to create 2-, 3-, and 4-chamber and short-axis (SAX) stacks for each patient with a 3 mm slice thickness and increment. As previously shown, a dedicated application (QMass Medis, Medis medical Imaging, Leiden, the Netherlands) was utilized to semi-automatically trace the endocardial border for the long-axis (LA) stacks (2-, 3-, 4-chamber stacks) for each phase of the cardiac cycle and both the endocardial and epicardial border for the end-systolic and end-diastolic phases in the SAX stack of the LV from the base to the apex. Papillary muscles were excluded from the endocardial contours in both the LA and SAX stacks and automatic segmentation was manually edited when necessary [5]. Another application (Qstrain Medis, Medis medical Imaging, Leiden, the Netherlands) was finally used to automatically calculate LV endocardial global longitudinal strain (GLS), endocardial global circumferential strain (GCS), and global radial strain (GRS). Endocardial LV-GLS and LV-GCS were averaged across the 2-, 3-, and 4-chamber stacks, while LV-GRS was assessed in basal (at mitral valve level), mid-ventricular (at papillary muscles level) and apical slices from the SAX stack.

### 2.4. ECV Analysis

For the ECV analysis, virtual monoenergetic images at 65 keV and at a fixed RR interval of 280 ms were reconstructed with a 1.5 mm slice thickness and 1 mm increment from the CCTA and the LE scan. In addition, the LE scan was used to generate spectral iodine images with a 1.5 mm slice thickness and 1 mm increment. Quantum iterative reconstruction (QIR) level 3 and the reconstruction kernel Qr40 were utilized as previously reported [16]. ECV analysis was performed with a prototype software (CT Cardiac Functional Analysis, syngo.via VB 60; Siemens) by another blinded member of the study team with 4 years of experience in cardiac imaging (V.M.). In more detail, the ECV calculation was based on the spectrally derived iodine concentration of the blood pool and the myocardium from the LE scan according to the following formula:

(1) $$ECV=\left(1-Hct\right)\;\cdot \;\frac{\left[{Iodine}_{myocardium}\right]}{\left[{Iodine}_{bloodpool}\right]}$$

A three-dimensional (3D) analysis was performed, matching a heart model generated from the CCTA data. The LV heart model was overlaid onto the corresponding ECV data and results were exported numerically in a 17-segments polar map. An ECV calculation was performed for the mid-myocardial compartment, defined as the myocardium between the inner 25% and outer 25% of the cardiac muscle.

### 2.5. Aortic Valve Calcification

Aortic valve calcification scoring was performed using a commercially available software (CaScore, syngo.via VB 60; Siemensstr. 3, 91301 Forchheim, Germany) according to the Agatston method [20].

### 2.6. Statistical Analysis

Variables were tested with the Shapiro–Wilk test for normal distribution. Variables are presented as means ± standard deviations when normally distributed and as medians and interquartile ranges when non-normally distributed. Categorical variables are reported as counts and percentages. The results of the strain analyses (LV-GLS, LV-GCS, and LV-GRS), ECV values, aortic valve calcification scores, and CT-derived left ventricular ejection fraction (EF) were compared using linear regression models, Student’s *t*-tests, Wilcoxon signed rank tests, and Fisher exact tests, as appropriate. A two-tailed *p* value below 0.05 was considered to infer statistical significance. Analyses were performed using R statistical software (R, version 4.3.2; R Foundation).

## 3. Results

### 3.1. Patient Characteristics

A total of 77 patients with severe AS were included in the study (28 females; 49 males; mean age 81 ± 8 years; mean body mass index 27 ± 5 kg/m^2^). A study flow-chart is presented in Figure 1. Patients had a median aortic valve calcification score of 2470 Agatston units (interquartile range (IQR), 1740, 3656 Agatston units) and a median left ventricular EF of 64% (IQR, 46%, 73%). Patients’ demographics are detailed in Table 1. Eleven patients (11/77 patients, 14%) presented with low-flow, low-gradient AS according to echocardiographic findings.

The cohort included subjects both without (67/77 patients, 87%) and with known myocardial disease (10/77 patients, 13%). Patients with known myocardial diseases had previous myocardial infarction (6/10 patients, 60%) or were diagnosed with cardiac amyloidosis (4/10 patients, 40%).

### 3.2. Overall Patient Sample

**Figure 2:**
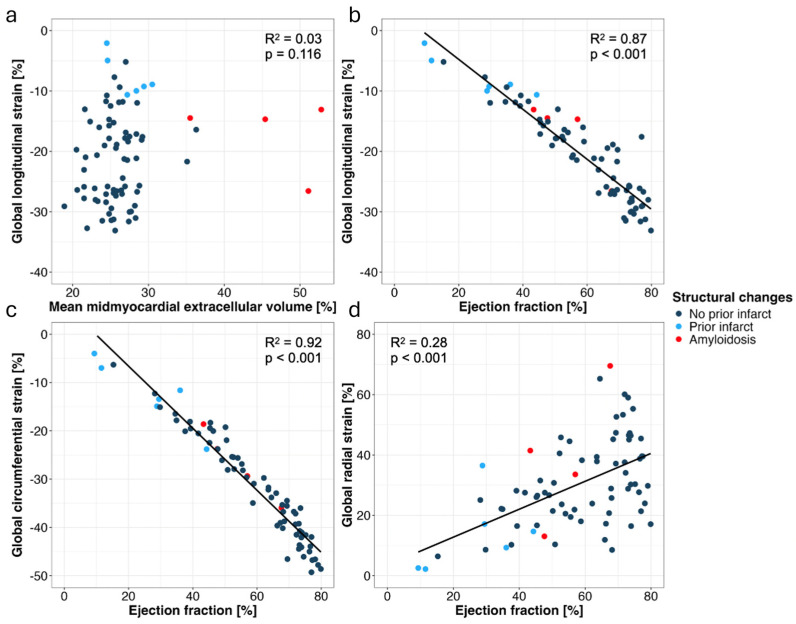
Regression analyses of global longitudinal strain (GLS) and mean mid-myocardial extracellular volume (ECV) (**a**) of GLS and CT-derived left ventricular ejection fraction (EF) (**b**), of global circumferential strain (GCS) and CT-derived left ventricular EF (**c**), and of global radial strain (GRS) and CT-derived left ventricular EF (**d**).

There was no significant correlation between GLS (Figure 2a), GCS, and GRS and mean mid-myocardial ECV (all, *p* > 0.05). Significant correlations were found between GLS, GCS, GRS, and CT-derived left ventricular EF (all, *p* < 0.001) (Figure 2b–d). No significant correlations were found between aortic valve calcification scores and mid-myocardial ECV, GLS, GCS, and GRS (all, *p* > 0.05).

### 3.3. Patients with Known Myocardial Disease

Patients with previous myocardial infarction showed lower GLS (*p* < 0.001), lower GCS (*p* < 0.001), and lower GRS values (*p* = 0.02) as well as a lower left ventricular EF (*p* < 0.001), compared with patients without myocardial disease, as summarized in Table 2.

Patients with cardiac amyloidosis showed higher mean mid-myocardial ECV values (median, 48.2%, IQR, 42.9%, 51.5%) compared with those without myocardial disease (median, 25.5%, IQR, 24.3%, 27.1%, *p* = 0.048).

**Figure 3:**
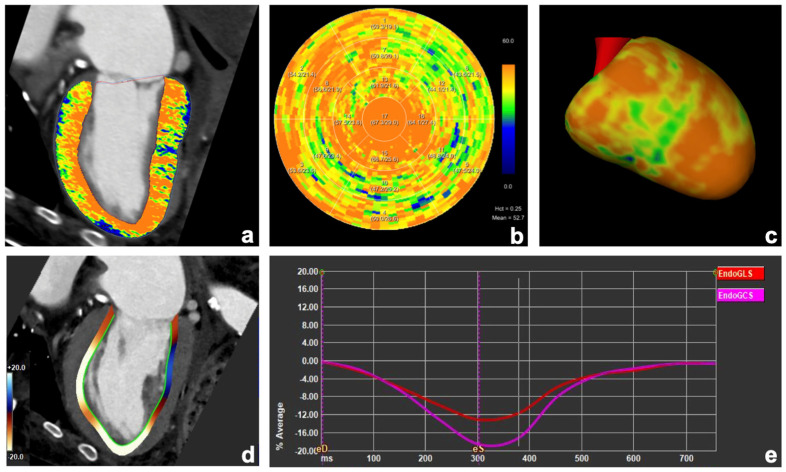
Images from an 83-year-old male patient with severe aortic stenosis and transthyretin cardiac amyloidosis (ATTR) showing a severe and diffuse increase in myocardial extracellular volume (ECV) values. ECV maps were calculated from the late enhancement scan using the spectral method with a hematocrit of 25% (**a**–**c**). Endocardial global longitudinal strain decreased (13%; (**d**,**e**)).

**Figure 4:**
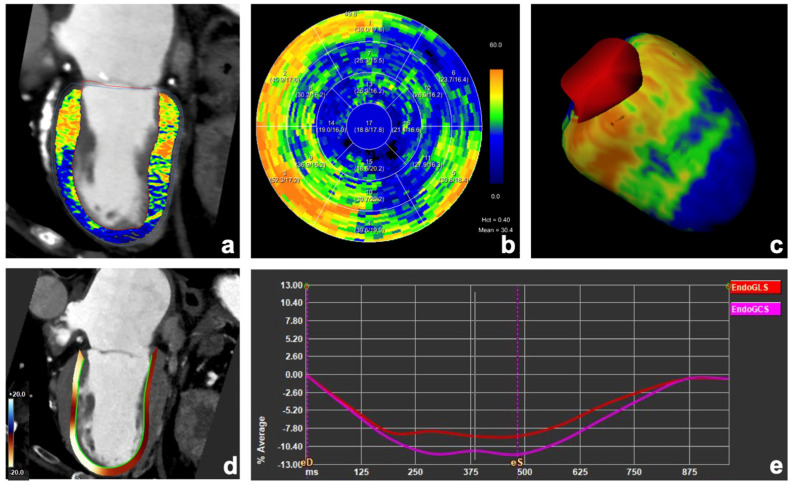
Images from a 94-year-old male patient with severe aortic stenosis showing an increase in myocardial extracellular volume (ECV) values in basal anterior, septal and infero-septal, and in the basal infero-lateral left ventricular wall. ECV maps were calculated from late enhancement scan using the spectral method with a hematocrit of 39% (**a**–**c**). Aneurysmatic dilation of the left ventricular apex (**a**,**d**) was present, along with a decreased endocardial global longitudinal strain of −9% (**d**,**e**).

Representative examples of images from a patient with severe AS and transthyretin cardiac amyloidosis and a patient with previous myocardial infarction are shown in Figure 3 and Figure 4, respectively.

### 3.4. Patients Without Known Myocardial Disease

In total, 67 patients without myocardial disease were included, among which 11 (11/67 patients, 16%) presented with low-flow, low-gradient AS and a reduced EF.

**Figure 5:**
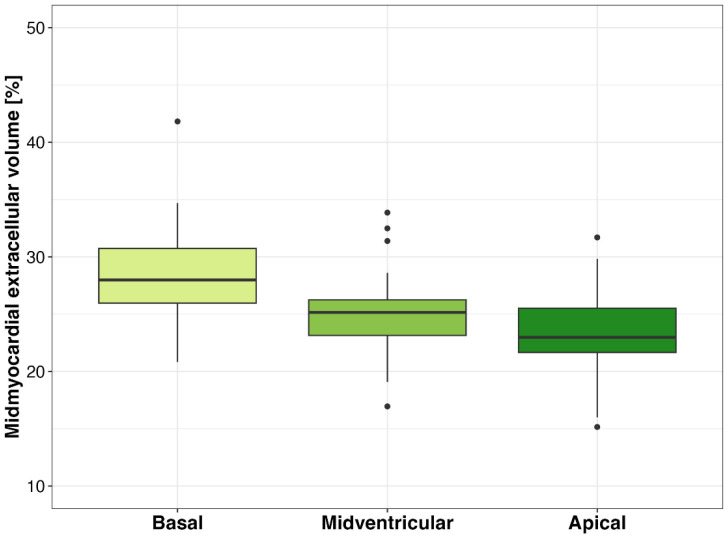
Box plots of extracellular volume (ECV) at basal, midventricular, and apical levels, showing the basal-to-apical ECV gradient in patients without known myocardial disease.

Significant differences were found between the mean mid-myocardial ECV at the basal (median, 28.0%, IQR, 26.0%, 30.7%), mid-ventricular (median, 25.1%, IQR, 23.1%, 26.3%), and apical levels (median 23.0%, IQR, 21.7%, 25.5%) in patients without known myocardial disease as shown in Figure 5 (all, *p* < 0.001). A regression analysis revealed a correlation between the basal–apical mid-myocardial ECV gradient and GLS (R = 0.28, *p* = 0.02).

**Figure 6:**
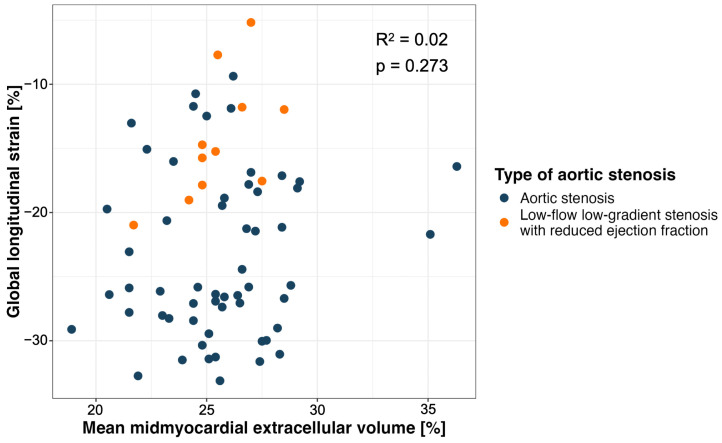
Scatter plot of global longitudinal strain (GLS) and mean mid-myocardial extracellular volume (ECV) in patients without known myocardial disease or cardiac amyloidosis. GLS values were lower in patients with low-flow, low-gradient aortic stenosis with reduced ejection fraction compared to patients with high-gradient aortic stenosis.

Patients with low-flow, low-gradient AS with a reduced EF showed significantly lower GLS values (median, −15.2%, IQR, −17.7%, −11.9%) than patients with high-gradient AS (median, −25.8%, IQR, −28.3%, −18.3%, *p* < 0.001). No significant correlation was found between GLS and the mean mid-myocardial ECV (*p* = 0.27) in patients with low-flow, low-gradient AS (Figure 6).

## 4. Discussion

This exploratory study evaluated the relationship between myocardial strain and ECV in patients with severe AS using advanced PCD-CT imaging techniques. In the overall patient sample, significant correlations were identified between GLS, GCS, GRS, and left ventricular EF (all, *p* < 0.001). Patients with cardiac amyloidosis had higher mid-myocardial ECV values, while patients with previous myocardial infarction showed reduced GLS (*p* < 0.05 for all). In patients without cardiac amyloidosis or prior myocardial infarction, a basal–apical mid-myocardial ECV gradient correlated with the GLS values (*p* < 0.05). Patients with low-flow, low-gradient AS and a reduced EF exhibited notably lower GLS values compared to those with high-gradient AS (*p* < 0.001).

Myocardial ECV as well as myocardial strain assessments are both validated tools to detect concomitant cardiac amyloidosis in patients with severe AS [21,22]. Our findings underscore the potential of CT-derived ECV as a non-invasive biomarker for cardiac amyloidosis in the context of AS, despite the fact that our study included only a low number of patients with cardiac amyloidosis. Previously, Scully et al. showed that CT-based ECV allows patients with AS and concomitant cardiac amyloidosis to be identified and allows both their risk to be stratified and their prognosis to be predicted [23]. These findings were recently confirmed by Patel et al. who further proved that patients with AS and cardiac amyloidosis demonstrated significantly higher CT-based ECV values compared with a healthy control population (37.4% versus 26.1%, *p* < 0.001) [24].

We found significant correlations between GLS, GCS, GRS, and CT-derived left ventricular EF (all, *p* < 0.001), with lower values observed in patients with prior myocardial infarctions, reflecting the well-known impact of a previous ischemic injury on myocardial function [25]. These results suggest that CT-based strain analysis could serve as a valuable tool for detecting and quantifying myocardial dysfunction in AS patients with cardiac comorbidities. No significant strain pattern was detected in the patient subgroup with cardiac amyloidosis. In contrast to our data, Bernhard et al. found a good diagnostic performance of myocardial strain analysis for detecting concomitant cardiac amyloidosis in patients with AS [22]. A possible explanation for this discrepancy is the limited sample size of patients with cardiac amyloidosis in our cohort, which may have reduced the statistical power to detect such patterns. Additionally, the variability in strain measurement reproducibility could have influenced our results, particularly given the known challenges with inter- and intra-reader agreement in strain assessments.

A recent CT-based study showed variable inter- and intra-reader agreement for left ventricular strain assessment with CT, which was largest for GLS and smallest for GRS [5]. We did not observe correlations between aortic valve calcification scores and ECV or myocardial strain, respectively. These results may reflect a selection bias as we mostly included patients with high grade AS and hence, with larger aortic valve calcium load.

In patients with low-flow, low-gradient AS, and a reduced ejection fraction, lower CT-derived GLS values were observed compared with those with high-gradient AS, confirming echocardiographic studies showing that longitudinal function calculated as GLS is more severely impaired in low-flow, low-gradient AS than in subjects with high-gradient AS [26]. This highlights the importance of GLS as a sensitive marker for identifying subtle myocardial impairment in this specific subgroup, which often presents a diagnostic and therapeutic challenge [27]. The observed basal–apical gradient in mid-myocardial ECV is in line with a recent CT study, where an increased ECV at the ventricular base values has different possible explanations: the ventricular base may be more prone to amyloid deposition and increased myocyte death, it may be characterized by less diversity of myocytes and matrix orientation, or it may be the main site of myocardial contractility alterations, leading to myocytes loss [24].

The following limitations of our study merit consideration. First, this was a single-center retrospective study including a limited number of patients. Second, both the strain and the ECV analysis software were limited to a single vendor, despite other software tools being available for these purposes as well. Third, the results were obtained in a selected group of patients with severe AS who were candidates for TAVR and who underwent ECG-gated retrospective spiral CT coronary angiography and LE imaging; further data are necessary to test the correlation of ECV and strain with functional and structural parameters in patients with less severe AS grades. Moreover, a relatively large number of patients were excluded from the final analysis due to technical reasons and metal artifacts, possibly introducing a selection bias and further hampering the external validity of the results. Finally, no outcome data were collected in this exploratory study, precluding prognostic considerations.

## 5. Conclusions

Advanced cardiac assessment with PCD-CT, including ECV calculation and myocardial strain measurements, has the potential to identify subjects with cardiac amyloidosis and reduced myocardial contractility within patient groups with aortic stenosis prior to valve replacement. The integration of CT-derived biomarkers, such as ECV and strain, into the evaluation process has the potential to refine TAVR decision-making by identifying patients at higher risk due to underlying myocardial disease. These biomarkers could also help predict post-TAVR outcomes, enabling personalized treatment strategies and closer monitoring for patients with impaired myocardial function or cardiac amyloidosis

## Figures and Tables

**Table 1:** Patients’ demographics.

Characteristic	*n* = 77 Patients
Sex	
Female	28 (36%)
Male	49 (64%)
Age [years]	81 ± 8
Body weight [kg]	75 ± 16
Body mass index [kg/m^2^]	27 ± 5
Average heart rate during data acquisition [bpm]	76 ± 15
Average hematocrit (%)	39 ± 0.05
Aortic valve calcium score derived by CT [Agatston units] *	2470 (1740–3656)
Average EF derived by echocardiography (%)	64 (46, 73)
Medical history	
Arterial hypertension	67 (87%)
Dyslipidemia	50 (65%)
Smoking history	24 (31%)
Diabetes	21 (27%)
Prior coronary revascularization	18 (23%)
Chronic kidney disease	21 (27%)
COPD	7 (9%)

Note: Unless otherwise indicated, data are the mean ± standard deviation or number of patients with percentages in parentheses. * Data are presented as median and interquartile range. bpm = beats per minute; COPD = chronic obstructive pulmonary disease; EF = ejection fraction; *n* = number of patients.

**Table 2:** Comparison of strain results in patients without and with previous myocardial infarction.

Parameter	Patients with Previous Myocardial Infarction	Patients Without Myocardial Diseases	*p*-Value
GLS (%)	−9.1 (−9.8, −5.9)	−21.7 (−27.6, −17.0)	<0.001
GCS (%)	−12.5 (−14.5, −8.1)	−35.3 (−42.0, −25.4)	<0.001
GRS (%)	12.0% (4.2, 16.5)	28.9 (21.7, 39.6)	0.02
LVEF (%)	29.1 (15.9, 34.4)	67.3 (50.7, 73.7)	<0.001

Note: Unless otherwise indicated, data are median ± IQR. GLS: global longitudinal strain; GCS: global circumferential strain; GRS: global radial strain; LVEF: left ventricular ejection fraction.

## Data Availability

Data are available for any needed verification.

## References

[1] Timmis A., Vardas P., Townsend N., Torbica A., Katus H., De Smedt D., Gale C.P., Maggioni A.P., Petersen S.E., Huculeci R. (2022). European Society of Cardiology: Cardiovascular disease statistics 2021: Executive Summary. Eur. Heart J. Qual. Care Clin. Outcomes.

[2] Osnabrugge R.L.J., Mylotte D., Head S.J., Van Mieghem N.M., Nkomo V.T., LeReun C.M., Bogers A.J.J.C., Piazza N., Kappetein A.P. (2013). Aortic stenosis in the elderly: Disease prevalence and number of candidates for transcatheter aortic valve replacement: A meta-analysis and modeling study. J. Am. Coll. Cardiol..

[3] Stens N.A., van Iersel O., Rooijakkers M.J.P., van Wely M.H., Nijveldt R., Bakker E.A., Rodwell L., Pedersen A.L.D., Poulsen S.H., Kjønås D. (2023). Prognostic Value of Preprocedural LV Global Longitudinal Strain for Post-TAVR-Related Morbidity and Mortality: A Meta-Analysis. JACC Cardiovasc. Imaging.

[4] Lee H.-J., Lee H., Kim S.M., Park J.-B., Kim E.K., Chang S.-A., Park E., Kim H.-K., Lee W., Kim Y.-J. (2020). Diffuse myocardial fibrosis and diastolic function in aortic stenosis. JACC Cardiovasc. Imaging.

[5] Bernhard B., Grogg H., Zurkirchen J., Demirel C., Hagemeyer D., Okuno T., Brugger N., De Marchi S., Huber A.T., Berto M.B. (2022). Reproducibility of 4D cardiac computed tomography feature tracking myocardial strain and comparison against speckle-tracking echocardiography in patients with severe aortic stenosis. J. Cardiovasc. Comput. Tomogr..

[6] He X., Li Y., Wang Y., Tian W., Li Z., Ge L., Wang G., Chen Z. (2024). Prognostic Value of CT-Derived Myocardial Biomarkers: Extracellular Volume Fraction and Strain in Patients with Severe Aortic Stenosis Undergoing Transcatheter Aortic Valve Replacement: A Systematic Review and Meta-analysis. Acad. Radiol..

[7] Bing R., Cavalcante J.L., Everett R.J., Clavel M.-A., Newby D.E., Dweck M.R. (2019). Imaging and impact of myocardial fibrosis in aortic stenosis. JACC Cardiovasc. Imaging.

[8] Abecasis J., Lopes P., Santos R.R., Maltês S., Guerreiro S., Ferreira A., Freitas P., Ribeiras R., Andrade M.J., Manso R.T. (2023). Prevalence and significance of relative apical sparing in aortic stenosis: Insights from an echo and cardiovascular magnetic resonance study of patients referred for surgical aortic valve replacement. Eur. Heart J. Cardiovasc. Imaging.

[9] Abecasis J., Lopes P., Maltes S., Santos R.R., Ferreira A., Ribeiras R., Andrade M.J., Uva M.S., Gil V., Félix A. (2024). Histopathological myocardial changes in patients with severe aortic stenosis referred for surgical valve replacement: A cardiac magnetic resonance correlation study. Eur. Heart J. Cardiovasc. Imaging.

[10] Blanke P., Weir-McCall J.R., Achenbach S., Delgado V., Hausleiter J., Jilaihawi H., Marwan M., Norgaard B.L., Piazza N., Schoenhagen P. (2019). Computed tomography imaging in the context of transcatheter aortic valve implantation (TAVI)/transcatheter aortic valve replacement (TAVR): An expert consensus document of the Society of Cardiovascular Computed Tomography. J. Cardiovasc. Comput. Tomogr..

[11] Francone M., Budde R.P.J., Bremerich J., Dacher J.N., Loewe C., Wolf F., Natale L., Pontone G., Redheuil A., Vliegenthart R. (2020). CT and MR imaging prior to transcatheter aortic valve implantation: Standardisation of scanning protocols, measurements and reporting-a consensus document by the European Society of Cardiovascular Radiology (ESCR). Eur. Radiol..

[12] Lisi C., Moser L.J., Mergen V., Klambauer K., Uçar E., Eberhard M., Alkadhi H. (2024). Advanced myocardial characterization and function with cardiac CT. Int. J. Cardiovasc. Imaging.

[13] Oyama-Manabe N., Oda S., Ohta Y., Takagi H., Kitagawa K., Jinzaki M. (2024). Myocardial late enhancement and extracellular volume with single-energy, dual-energy, and photon-counting computed tomography. J. Cardiovasc. Comput. Tomogr..

[14] Muthalaly R.G., Tan S., Nelson A.J., Abrahams T., Han D., Tamarappoo B.K., Dey D., Nicholls S.J., Lin A., Nerlekar N. (2024). Variation of computed tomography-derived extracellular volume fraction and the impact of protocol parameters: A systematic review and meta-analysis. J. Cardiovasc. Comput. Tomogr..

[15] Lacaita P.G., Luger A., Troger F., Widmann G., Feuchtner G.M. (2024). Photon-Counting Detector Computed Tomography (PCD-CT): A New Era for Cardiovascular Imaging? Current Status and Future Outlooks. J. Cardiovasc. Dev. Dis..

[16] Mergen V., Sartoretti T., Klotz E., Schmidt B., Jungblut L., Higashigaito K., Manka R., Euler A., Kasel M., Eberhard M. (2022). Extracellular Volume Quantification with Cardiac Late Enhancement Scanning Using Dual-Source Photon-Counting Detector CT. Investig. Radiol..

[17] Aquino G.J., O’Doherty J., Schoepf U.J., Ellison B., Byrne J., Fink N., Zsarnoczay E., Wolf E.V., Allmendinger T., Schmidt B. (2023). Myocardial Characterization with Extracellular Volume Mapping with a First-Generation Photon-counting Detector CT with MRI Reference. Radiology.

[18] Emoto T., Oda S., Kidoh M., Nakaura T., Nagayama Y., Sakabe D., Kakei K., Goto M., Funama Y., Hatemura M. (2021). Myocardial Extracellular Volume Quantification Using Cardiac Computed Tomography: A Comparison of the Dual-energy Iodine Method and the Standard Subtraction Method. Acad. Radiol..

[19] Baumgartner H., Falk V., Bax J.J., De Bonis M., Hamm C., Holm P.J., Iung B., Lancellotti P., Lansac E., Rodriguez Muñoz D. (2017). ESC Scientific Document Group 2017 ESC/EACTS Guidelines for the management of valvular heart disease. Eur. Heart J..

[20] Eberhard M., Hinzpeter R., Polacin M., Morsbach F., Maisano F., Nietlispach F., Nguyen-Kim T.D.L., Tanner F.C., Alkadhi H. (2019). Reproducibility of aortic valve calcification scoring with computed tomography—An interplatform analysis. J. Cardiovasc. Comput. Tomogr..

[21] Scully P.R., Patel K.P., Saberwal B., Klotz E., Augusto J.B., Thornton G.D., Hughes R.K., Manisty C., Lloyd G., Newton J.D. (2020). Identifying cardiac amyloid in aortic stenosis: ECV quantification by CT in TAVR patients. JACC Cardiovasc. Imaging.

[22] Bernhard B., Leib Z., Dobner S., Demirel C., Caobelli F., Rominger A., Schütze J., Grogg H., Alwan L., Spano G. (2023). Routine 4D Cardiac CT to Identify Concomitant Transthyretin Amyloid Cardiomyopathy in Older Adults with Severe Aortic Stenosis. Radiology.

[23] Scully P.R., Patel K.P., Klotz E., Augusto J.B., Thornton G.D., Saberwal B., Haberland U., Kennon S., Ozkor M., Mullen M. (2022). Myocardial fibrosis quantified by cardiac CT predicts outcome in severe aortic stenosis after transcatheter intervention. JACC Cardiovasc. Imaging.

[24] Patel K.P., Scully P.R., Saberwal B., Sinha A., Yap-Sanderson J.J.L., Cheasty E., Mullen M., Menezes L.J., Moon J.C., Pugliese F. (2024). Regional distribution of extracellular volume quantified by cardiac CT in aortic stenosis: Insights into disease mechanisms and impact on outcomes. Circ. Cardiovasc. Imaging.

[25] Khan J.N., Singh A., Nazir S.A., Kanagala P., Gershlick A.H., McCann G.P. (2015). Comparison of cardiovascular magnetic resonance feature tracking and tagging for the assessment of left ventricular systolic strain in acute myocardial infarction. Eur. J. Radiol..

[26] Adda J., Mielot C., Giorgi R., Cransac F., Zirphile X., Donal E., Sportouch-Dukhan C., Réant P., Laffitte S., Cade S. (2012). Low-flow, low-gradient severe aortic stenosis despite normal ejection fraction is associated with severe left ventricular dysfunction as assessed by speckle-tracking echocardiography: A multicenter study. Circ. Cardiovasc. Imaging.

[27] D’Andrea A., Carbone A., Agricola E., Riegler L., Sperlongano S., Tocci G., Scarafile R., Formisano T., Capogrosso C., Cappelli Bigazzi M. (2019). Predictive Value of Left Ventricular Myocardial Deformation for Left Ventricular Remodeling in Patients With Classical Low-Flow, Low-Gradient Aortic Stenosis Undergoing Transcatheter Aortic Valve Replacement. J. Am. Soc. Echocardiogr..

